# Inhibition of MDA-MB-231 breast cancer cell migration and invasion activity by andrographolide via suppression of nuclear factor-κB-dependent matrix metalloproteinase-9 expression

**DOI:** 10.3892/mmr.2014.2872

**Published:** 2014-11-05

**Authors:** ZANJING ZHAI, XINHUA QU, HAOWEI LI, ZHENGXIAO OUYANG, WEI YAN, GUANGWANG LIU, XUQIANG LIU, QIMING FAN, TINGTING TANG, KERONG DAI, AN QIN

**Affiliations:** 1 Shanghai Key Laboratory of Orthopaedic Implants, Department of Orthopaedics, Ninth People’s Hospital, Shanghai Jiao Tong University School of Medicine, Shanghai 200011, P.R. China; 2 Department of Orthopaedics, Affiliated Tumor Hospital of Xiangya School of Medicine, Central South University, Changsha, Hunan 410013, P.R. China; 3 Department of Orthopaedics, Wendeng Zhenggu Hospital of Shandong Province, Weihai, Shandong 264400, P.R. China; 4 Department of Orthopedic Surgery, The Central Hospital of Xuzhou, Affiliated Hospital of Medical College of Southeast University, Xuzhou, Jiangsu 221009, P.R. China

**Keywords:** breast cancer, MBA-MD-231, andrographolide, invasion, matrix metalloproteinase-9, nuclear factor κB

## Abstract

Breast cancer is one of the most common types of cancer worldwide. The majority of patients with cancer succumb to the disease as a result of distant metastases (for example, in the bones), which cause severe complications. Despite advancements in breast cancer treatment, chemotherapeutic outcomes remain far from satisfactory, prompting a search for effective natural agents with few side-effects. Andrographolide (AP), a natural diterpenoid lactone isolated from *Andrographis paniculata*, inhibits cancer cell growth. The current study aimed to examine the effect of AP on breast cancer cell proliferation, survival and progression *in vitro* and also its inhibitory activity on breast cancer bone metastasis *in vivo.* To achieve this, CCK8, flow cytometry, migration, invasion, western blot, PCR and luciferase reporter assay analyses were performed *in vitro* as well as establishing intratibial xenograft model of breast cancer bone metastasis *in vivo*. The results demonstrated that AP inhibits the migration and invasion of the MBA-MD-231 aggressive breast cancer cell line at non-lethal concentrations, in addition to suppressing proliferation and inducing apoptosis at high concentrations *in vitro*. *In vivo*, AP significantly inhibited the growth of tumors planted in bone and attenuated cancer-induced osteolysis. Tartrate-resistant acid phosphatase staining revealed osteoclast activation in tumor-bearing mice and AP was observed to attenuate this activation. The anti-tumor activity of AP *in vitro* and *in vivo* correlates with the downregulation of the nuclear factor κB signaling pathway and the inhibition of matrix metalloproteinase-9 expression levels. These results indicate that AP may be an effective anti-tumor agent for the treatment of breast cancer bone metastasis.

## Introduction

Breast cancer bone metastasis causes serious complications, including chronic pain and pathologic fractures, which severely reduce quality of life (1). Bone metastasis secondary to breast cancer is associated with a poor prognosis (2) and current therapies for the management of metastasis and osteolysis are far from satisfactory. Hence, it is necessary to develop novel alternative therapies with improved efficacy and fewer side-effects.

Metastasis is a complicated process, which proceeds through a sequence of cancer cell proliferation, adhesion, invasion and migration (3,4). Matrix metalloproteinases (MMPs) are thought to be critical to this process (5–7) and MMP-9 is considered to be the most relevant for tumor invasion (8). MMP regulation occurs at multiple levels and a number of stimuli activate MMP-9, including growth factors, cytokines and phorbol 12-myristate 13-acetate (PMA) (9–11). The MMP-9 promoter contains multiple DNA binding sites for transcription factors, including nuclear factor κB (NF-κB) (12). Therefore, the downregulation of MMP-9 expression may be a useful strategy for tumor metastasis intervention. Plant-derived compounds with a chemopreventive potential have been shown to inhibit the invasiveness of several types of cancer by modifying MMP-9 expression (13,14).

Andrographolide (AP) is a diterpenoid lactone isolated from the traditional Chinese and Indian medicinal plant *Andrographis paniculata* and it is widely used for its efficacy and favorable safety profile in a number of diseases (15,16). AP has gained attention for its anticancer (17,18), anti-inflammation (19,20), hepatoprotection (21,22) and anti-infection (16) activities. Previous studies have demonstrated the anti-cancer effect of AP in the MCF-7 and TD-47 breast cancer cell lines (23–25); however, the effect of AP on the more aggressive MDA-MB-231 cancer cell line and on breast cancer bone metastasis *in vivo* has not been reported.

The aim of the present study was to identify supplementary therapeutic strategies for the treatment of breast cancer metastasis and osteolysis through the investigation of the *in vitro* action of AP on the invasion and migration of MDA-MB-231 cells. In addition, the efficacy of AP in the prevention of breast cancer bone metastasis and osteolysis were investigated in an *in vivo* mouse xenograft model.

## Materials and methods

### Media and reagents

AP and PMA were purchased from Sigma-Aldrich (St. Louis, MO, USA). Minimum Essential Medium-α (α-MEM), fetal bovine serum (FBS) and penicillin were obtained from Gibco-BRL (Gaithersburg, MD, USA). The Cell Counting kit (CCK)-8 assay was purchased from Dojindo Molecular Technology (Tokyo, Japan). Primary antibodies (monoclonal rabbit antibody; species reactivity, human) for β-actin, phospho-IκBα, IκBα and MMP-9 were purchased from Cell Signaling Technology, Inc. (Beverly, MA, USA). The Luciferase Assay system was from Promega (Sydney, Australia). Tris, glycine, NaCl, SDS, and other reagents were from Sigma-Aldrich. The Vybrant^®^ Apoptosis Assay kit #2 was from Invitrogen (Carlsbad, CA, USA).

### Cell viability assay

MDA-MB-231 cells were cultured in L-15 Medium (Gibco Life Technologies, Beijing, China) with 10% FBS and maintained in a humidified atmosphere of 5% CO_2_ at 37°C. The complete medium was changed every other day. The cells were treated with increasing concentrations of AP (0, 7.5, 15, 30, 60 or 120 μM) for two days prior to the cell viability assays. The anti-proliferative effect of AP on MDA-MB-231 cells was assessed using CCK-8. Briefly, following treatment, 10 μl CCK-8 solution was added to each well and incubated for 4 h. The absorbance was measured at a wavelength of 450 nm using a ELX800 absorbance microplate reader (BioTek Instruments, Inc., Winooski, VT, USA) at a wavelength of 450 nm (reference, 650 nm). The effect of AP on cell viability was expressed as a percentage of cell viability, with the vehicle-treated control cells set as 100%.

### Apoptosis assay

AP induction of apoptosis in MBA-MD-231 cells was determined with the Vybrant^®^ Apoptosis Assay kit #2. Following treatment, cells were washed twice with cold phosphate-buffered saline (PBS) and resuspended in 1X Annexin-binding buffer. Early apoptosis was detected via staining with Alexa Fluor^®^ 488 Annexin V and propidium iodide. Fluorescence-activated cell sorting was performed using a FACScan™ flow cytometer and data were acquired using CellQuest software, version 3.0 (BD Biosciences, Sunnyvale, CA, USA).

### Migration assay

Transwell^®^ Permeable Supports (Corning Inc., Acton, MA, USA), 24-well chambers with 8-μm pore polycarbonate filters, were used as described by the manufacturer. MDA-MB-231 cells (5×10^4^) were placed in 100 μl serum-free medium in the presence or absence of AP and 600 μl complete medium with 80 nM PMA was placed into the lower wells. Following treatment, cells were fixed with 100% methanol for 20 min and stained with Trypan blue for 30 min. Non-migrating cells on the upper side of the filter were removed with cotton swabs. Migration was quantified by counting the number of cells on the lower surface of the filter.

### Invasion assay

BioCoat™ Matrigel™ Invasion Chamber (BD Biosciences), 24-well chambers with 8-μm pore polycarbonate filters, were used according to the manufacturer’s instructions. MDA-MB-231 cells (5×10^4^) were placed in 100 μl serum-free medium in the presence or absence of AP, and 600 μl complete medium with 80 nM PMA was placed in the lower wells. Following treatment, cells on the upper side of the filters were removed. Invading cells on the underside of the filter were fixed with 100% methanol for 2 min and stained with Liu’s stain for 2 min. Invasion was quantified by counting the number of cells on the lower surface of the filter.

### Intratibial xenograft model of breast cancer bone metastasis

BALB/c nu/nu mice (Harlan, Indianapolis, IN, USA) were housed in individual cages, maintained in an animal facility under controlled temperature (22–24°C) and humidity (50–60%) conditions and a 12 h light/dark cycle with free access to food and water. Cultured MDA-MB-231 cells were resuspended in PBS at a density of 5×10^6^ cells/ml (26,27). An aliquot (10 μl) of the cell suspension was slowly injected through the anterior tuberosity of the proximal tibia in the right limbs of 5- to 6-week-old female BALB/c nu/nu mice (Harlan, Indianapolis, IN, USA). The mice were randomly assigned to vehicle (0.9% NaCl, n=8) or AP (50 mg/kg body weight vehicle, n=8) groups and treated via an intraperitoneal injection every other day. After 28 days, a bioluminescence assay was performed and fluorescence intensity was quantified (Living Image v3.2, Caliper; Caliper Life Sciences, Hopkinton, MA, USA). Radiographs using the Directview Vita CR system. (Carestream Kodak, Rochester, NY, USA) of the tibiae were obtained prior to euthanasia with ketamine, administered by intraperitoneal injection (0.8 ml/100 g body weight). The product from Carestream Kodak was. Tissues were removed and fixed in 4% paraformaldehyde for 1 day at 4°C followed by decalcification in 12% EDTA. Decalcified bones were paraffin-embedded and sectioned. Samples were subjected to tartrate-resistant acid phosphatase (TRAP) staining to identify osteoclasts on the bone surface. Immunostaining for Ki67 (Dako, Carpinteria, CA, USA) and terminal deoxynucleotidyl transferase-mediated dUTP nick-end labeling (TUNEL) were performed as previously described (28,29). Ki67- and TUNEL-positive tumor cells were counted and the percentages of positive cells were calculated. This study was approved by the ethics committee of Shanghai Ninth People’s Hospital Affiliated to Shanghai Jiao Tong University School of Medicine (Shanghai, China).

### RNA isolation and reverse transcription-quantitative polymerase chain reaction (RT-qPCR)

RNA isolation was performed as previously described (30). Total RNA was extracted using the Qiagen RNeasy Mini kit (Qiagen, Valencia, CA, USA) following the manufacturer’s instructions. cDNA was synthesized from 1 mg of total RNA using reverse transcriptase (TaKaRa Biotechnology, Otsu, Japan). MMP-9 transcript expression levels were determined using the MiniOpticon Real-Time PCR system (Bio-Rad Laboratories, Hercules, CA, USA). qPCR was performed in a thermocycler (Biometra, T-Gradient Thermoblock, Germany) with a reaction volume of 10 μl containing 0.03 μg complementary DNA product, 2 μM forward and reverse primers and the KAPA™ SYBR^®^ FAST qPCR reagent (Kapa Biosystems, Wilmington, MA, USA). The primers used were as follows: Forward, 5′-GAACCAATCTCACCGACAGG-3′, and reverse, 5′-GCCACCCGAGTGTAACCATA-3′ for MMP-9; and forward, 5′-TCTGCTGGAAGGTGGACAGT-3′, and reverse, 5′-CCTCTATGCCAACACAGTGC-3′ for β-actin. Cycling conditions were as follows: 40 cycles of 95°C for 5 sec and 60°C for 34 sec. β-actin was included as a reference control. The comparative 2^−ΔΔCt^ method was used to calculate the relative expression of each gene (30).

### NF-κB-dependent luciferase reporter assay

The effect of AP on PMA-induced NF-κB activation was measured in MDA-MB-231 cells stably transfected with an NF-κB luciferase reporter construct (13). MDA-MB-231 cells were maintained in serum-free medium for 12 h, pretreated with AP for 1 h, followed by stimulation with PMA for 20 h. Subsequently, the cell lysis was incubated with substrate (Promega, Madison, WI, USA) at room temperature for about 2min, luciferase activity was measured using the Promega Luciferase Assay System (Promega, Madison, WI, USA). Luciferase activity was measured and normalized to the internal control. Results were obtained from three independent experiments.

### Western blotting

Western blotting was performed as previously described (30). The vehicle- or AP-treated cells were pretreated with PMA, washed twice in PBS and lysed in ice-cold lysis buffer (50 mM Tris pH 7.5, 150 mM NaCl, 1% Nonidet P-40, 0.1% SDS, 1% sodium deoxycholate) supplemented with phenylmethanesulfonyl fluoride (Shen Neng Bo Cai Corp., Shanghai, China). Lysates were maintained on ice for 30 min followed by centrifugation at 12,000 × g for 10 min. Protein concentrations were determined using a bicinchoninic acid (BCA) assay (Thermo Scientific, Rockford, IL, USA). Equal amounts of protein were separated by 10% SDS-PAGE and electroblotted onto polyvinylidene fluoride membranes (Roche, Mannheim, Germany). The membranes were blocked with 5% (w/v) skim milk solution for 1 h and probed with primary antibodies (β-actin, 1:1,000; phospho-IκBa, 1:1,000; IκBa, 1:1,000; and MMP-9, 1:1,000) at room temperature for 4 h, followed by incubation with horseradish peroxidase-conjugated secondary antibodies (anti-human; Cell Signaling Technology, Inc.; 1:5,000) for 1 h. Antibody reactivity was visualized using an Odyssey^®^ Infrared Imaging system (Li-Cor, Lincoln, NE, USA).

### Statistical analysis

Significant differences were determined with the Student’s t-test using SPSS v13.0 software (SPSS Inc., Chicago, IL, USA). P<0.05 was considered to indicate a statistically significant difference.

## Results

### AP inhibits the proliferation of MDA-MB-231 breast cancer cells and promotes apoptosis at high concentrations

**Figure 1:**
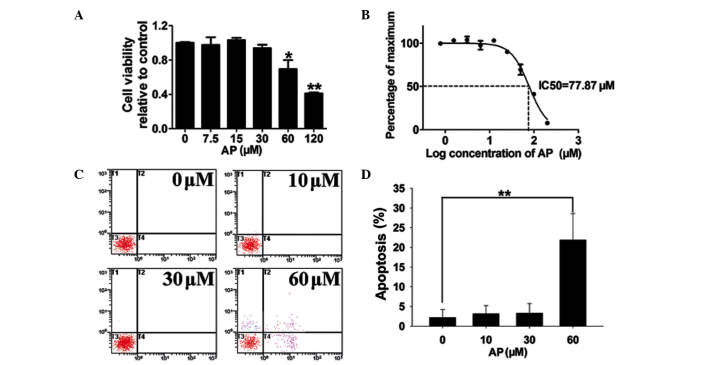
Andrographolide (AP) inhibits MDA-MB-231 breast cancer cell proliferation and promotes apoptosis at high concentrations. (A) Viability of AP-treated MDA-MB-231 cells. (B) The half-maximal inhibitory concentration (IC_50_) of AP was 77.87 μM. (C) Flow cytometric analysis of AP-treated MDA-MB-231 cells. (D) Percentage of apoptotic cells. Results are presented as the mean ± standard deviation of three independent experiments. ^*^P<0.05, ^**^P<0.01 vs. 0 μM.

Following a 48-h culture, a CCK-8 proliferation assay revealed that AP did not affect MDA-MB-231 cell proliferation at concentrations ≤30 μM (Fig. 1A). AP significantly suppressed cell proliferation at concentrations ≥60 μM. The calculated IC_50_ for AP is 77.87 μM (Fig. 1B). In cells treated with 10 or 30 μM AP, the observed apoptotic effects were similar to those of the vehicle control; however, the higher concentration of 60 μM AP induced apoptosis in 22% of cells (Fig. 1C and D). In order to exclude AP-mediated apoptosis, non-lethal concentrations (≤30 μM) were used in subsequent experiments.

### AP inhibits PMA-induced MDA-MB-231 cell migration and invasion in a concentration-dependent manner

**Figure 2:**
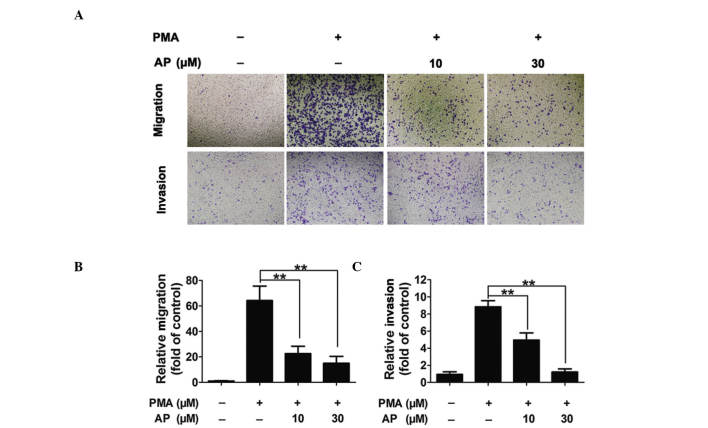
Andrographolide (AP) inhibits MDA-MB-231 breast cancer cell invasion and migration in a concentration-dependent manner at sub-lethal concentrations *in vitro*. (A) Membrane-associated, Liu-stained MDA-MB-231 breast cancer cells following treatment with AP and PMA. The numbers of (B) migrated and (C) invasive cells were counted. Results were recorded at least three times in three independent experiments and are presented as the mean ± standard deviation, n=3. ^*^P<0.05 and ^**^P<0.01.

PMA (80 nM) induced increased levels of MDA-MB-231 cell migration and invasion compared with those observed in the untreated cells; however, pretreatment with AP inhibited the PMA-induced migration and invasion in a concentration-dependent manner (Fig. 2A). Quantitative analysis confirmed AP inhibition of cell migration and invasion at concentrations as low as 10 μM (Fig. 2B and C).

### AP inhibits breast cancer bone metastasis and osteolysis in vivo

**Figure 3:**
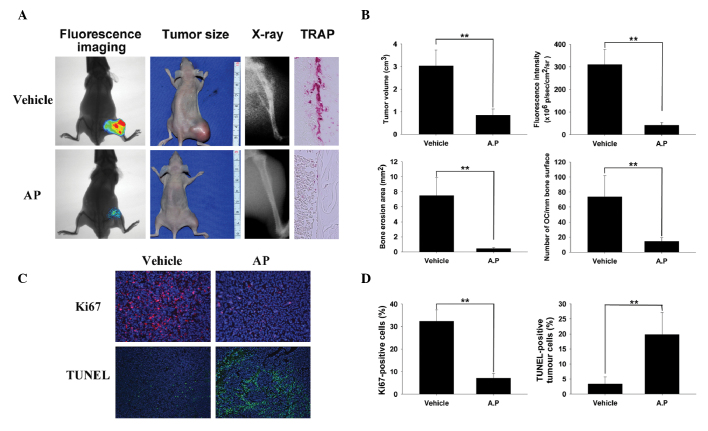
Bioluminescence of MDA-MB-231 human breast cancer cells in the anterior tuberosity of the proximal tibia in right limb of mice treated with vehicle (0.9% sodium chloride, n=8) or andrographolide (AP; 50 mg/kg body weight in vehicle, n=8) (A) The effects of AP on breast cancer-induced osteolytic lesions in mice as shown by X-ray imaging. Mouse tibia were collected from each group and sectioned for tartrate-resistant acid phosphatase (TRAP) staining (red signal). (B) Fluorescence intensity, tumor volume, bone erosion area, and number of osteoclasts (OC) were quantified and calculated. Experiments were performed in triplicate as three independent experiments. Results are presented as the mean ± standard deviation (SD), n=3. (C) Immunostaining for Ki67 and terminal deoxynucleotidyl transferase-mediated dUTP nick-end labeling (TUNEL). (D) Ki67- and TUNEL-positive tumor cells were counted and the percentages of positive cells were calculated. Experiments were performed in triplicate as three independent experiments and results are presented as the mean ± SD, n=3. ^*^P<0.05 and ^**^P<0.01.

To determine the effects of AP on breast cancer bone metastasis and cancer cell-induced osteolysis *in vivo*, a mouse xenotransplant model was used with human breast cancer cells (luciferase-labeled MDA-MB-231) (26,31). MDA-MB-231 cells were injected directly into the tibiae plateau via a percutaneous approach. After 28 days, bioluminescence was detected in the limbs of the control mice; however, the area and density of bioluminescence were reduced in the AP group compared with those in the control group (Fig. 3A), indicating that AP effectively suppressed breast cancer bone metastasis and growth *in vivo*. These observations were consistent with the results of the tumor volume assay (Fig. 3A). To confirm that osteolytic bone metastasis was blocked by AP, the osteolysis in the long bones of the hind legs was examined using radiography. AP significantly inhibited cancer cell-induced osteolysis (represented by radiolucency; Fig. 3A). TRAP staining (red) revealed numerous osteoclasts with intense activity in the vehicle-treated controls, however, in contrast, the number of osteoclasts was markedly reduced at the boundary in the treated mice (Fig. 3A), indicating that AP suppressed tumor-related osteolysis by inhibiting osteoclasts *in vivo*. All the results were confirmed using quantitative analysis (Fig. 3B). The proliferation-indicator Ki67 assay and the apoptosis-indicator TUNEL assay were also performed. Treatment of MDA-MB-231 tumor cells with AP (50 mg/kg) suppressed cellular proliferation compared with that in the control cells (Fig. 3C). The percentage of Ki67-positive cell nuclei was 7.1% in the AP-treated group and 32.4% in the vehicle-treated group (Fig. 3D). The levels of apoptosis were significantly increased in the AP-treated group of MDA-MB-231 cell-associated breast tumors compared with those of the vehicle-treated group in the TUNEL assay (Fig. 3C and D). All *in vivo* data were consistent with the *in vitro* data, demonstrating that AP inhibits MDA-MB-231 cancer cell invasion and migration and suppresses tumor-induced osteolysis, possibly via inhibited osteoclast activity.

### AP reduces PMA-stimulated MMP-9 secretion and expression

**Figure 4:**
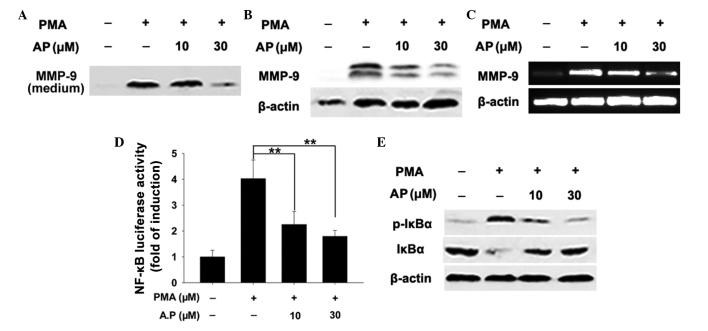
Andrographolide (AP) reduces phorbol 12-myristate 13-acetate (PMA)-stimulated matrix metalloproteinase (MMP)-9 expression via the suppression of the nuclear factor κB (NF-κB) signaling pathway. (A) Immunodetection of MMP-9 in the media of AP- and PMA-treated MDA-MB-231 cells. (B) Western blotting of MMP-9 in AP- and PMA-treated MDA-MB-231 cells. (C) MMP-9 gene expression in cells exposed to AP and PMA as in (B). (D) Luciferase activity (NF-κB expression) in stably transfected MDA-MB-231 cells treated with AP and PMA. Results were obtained from three independent experiments and are presented as the mean ± standard deviation. ^**^P<0.01. (E) Total and phosphorylated NF-κ-B inhibitor α (IκBα) in AP- and PMA-treated MDA-MB-231 whole cell lysates.

MMP-9 mediates tumor invasion and migration. In the current study, AP reduced the levels of MMP-9 secretion into the medium compared with those observed in the control cells (Fig. 4A). Consistent with the aforementioned findings, treatment of MDA-MB-231 cells with AP reduced PMA-stimulated MMP-9 protein expression in a concentration-dependent manner (Fig. 4B). qPCR revealed that PMA-induced MMP-9 mRNA expression decreased with AP treatment, indicating that AP-mediated inhibition of MMP-9 occurs at the transcriptional level (Fig. 4C).

### AP suppresses NF-κB signaling

MMP-9 is highly inducible in response to various stimuli and the MMP-9 promoter contains a binding site for transcription factor NF-κB (12), hence it can be used to detect NF-κB signaling. Measurement of NF-κB-dependent luciferase activity in MDA-MB-231 cells revealed that PMA-induced NF-κB transcriptional activity was suppressed by AP (Fig. 4D). NF-κB is normally sequestered in the cytoplasm in an inactive form associated with NF-κ-B inhibitor α (IκBα). Upon stimulation, the NF-κB subunit is released via the phosphorylation and proteasomal degradation of IκBa and translocated to the nucleus to initiate target gene transcription (32,33). AP was observed to prevent the PMA-induced degradation of IκBa (Fig. 4E). As degradation of IκBα is primarily the result of IκBα phosphorylation (33), it was hypothesized that this effect may be due to the AP-induced inhibition of IκBα phosphorylation. In the present study, AP caused a concentration-dependent reduction in PMA-induced IκBα phosphorylation (Fig. 4E). These results indicate that the inhibitory effect of AP on NF-κB signaling occurs via the inhibition of IκBa phosphorylation, which in turn suppresses transcriptional activity. NF-κB signaling activates MMP-9 transcription; thus, these results indicate that AP attenuates MMP-9 expression by inhibiting NF-κB signaling.

## Discussion

Previous studies have revealed the anti-cancer activity of AP (17,18). The current study investigated the utility of AP in fighting aggressive MDA-MB-231 breast cancer cell invasion and bone metastasis. It was revealed that AP effectively inhibits breast cancer cell migration and invasion *in vitro*. *In vivo*, AP inhibits breast cancer bone metastasis, suppresses tumor growth and induces tumor apoptosis in bone. This inhibition was associated with the downregulation of MMP-9 expression levels.

MMP-9 expression levels are highly correlated with breast cancer cell invasion (34) and agents that downregulate MMP-9 have been observed to inhibit tumor invasion (9,35). MMP-9 is inducible by a number of stimuli; the MMP-9 promoter contains DNA-binding sites for NF-κB, which regulates MMP-9 expression and secretion (36,37). The transcription factor NF-κB regulates the transcription of genes associated with cancer development, tumor invasion and inflammation. It is a target for numerous biologically active phytochemicals, including curcumin, resveratrol and epigallocatechin gallate. Exposure of cells to stimuli such as PMA leads to IκBa phosphorylation and degradation, allowing NF-κB to translocate to the nucleus where it binds to the MMP-9 promotor and activates transcription (9).

In the current study, AP was revealed to inhibit PMA-induced MMP-9 expression. The specific response of MMP-9 indicates that its downregulation by AP is mediated through an upstream event. Concurrently, PMA was observed to increase the levels of NF-κB transcriptional activity, whilst AP inhibited PMA-induced NF-κB transcriptional activity. These results confirm that NF-κB signaling is the molecular target for AP-induced inhibition of MMP-9 expression. Furthermore, the AP-induced reduction of PMA-stimulated NF-κB transcriptional activity was identified to be due to the inhibition of IκBa phosphorylation and IκBa proteasomal degradation. However, the mechanisms by which AP inhibits the phosphorylation of IκBa remain unclear.

In conclusion, AP-induced inhibition of IκBa phosphorylation was revealed to be the underlying mechanism of its effect on PMA-stimulated MDA-MB-231 cancer cell invasion. At sub-lethal concentrations, AP inhibits breast cancer cell migration and invasion via the downregulation of MMP-9 expression levels. The molecular mechanism by which AP inhibits MMP-9 expression involves the suppression of NF-κB activation. Tumor metastasis is often associated with poor prognosis and high mortality in breast cancer, prompting the requirement for the discovery and development of novel therapeutic strategies that target early tumor invasiveness and/or metastasis. AP reduces the invasiveness of highly aggressive MDA-MB-231 breast cancer cells *in vitro*, inhibits breast cancer bone metastasis, tumor growth, and tumor-induced osteolysis, and induces tumor apoptosis *in vivo*. It is thus a promising candidate therapeutic agent against breast cancer invasion and metastasis.
